# Use of *in vitro* methods combined with *in silico* analysis to identify potential skin sensitizers in the Tox21 10K compound library

**DOI:** 10.3389/ftox.2024.1321857

**Published:** 2024-02-28

**Authors:** Zhengxi Wei, Tuan Xu, Judy Strickland, Li Zhang, Yuhong Fang, Dingyin Tao, Anton Simeonov, Ruili Huang, Nicole C. Kleinstreuer, Menghang Xia

**Affiliations:** ^1^ National Institutes of Health, National Center for Advancing Translational Sciences, Bethesda, MD, United States; ^2^ Inotiv, Inc., Research Triangle Park, NC, United States; ^3^ National Toxicology Program Interagency Center for the Evaluation of Alternative Toxicological Methods, Division of the National Toxicology Program, National Institute of Environmental Health Sciences, Research Triangle Park, NC, United States

**Keywords:** quantitative high-throughput screening KeratinoSens, quantitative structure–activity screening modeling, defined approach, machine learning, skin sensitization, Tox21 10K compound library

## Abstract

**Introduction:** Skin sensitization, which leads to allergic contact dermatitis, is a key toxicological endpoint with high occupational and consumer prevalence. This study optimized several *in vitro* assays listed in OECD skin sensitization test guidelines for use on a quantitative high-throughput screening (qHTS) platform and performed *in silico* model predictions to assess the skin sensitization potential of prioritized compounds from the Tox21 10K compound library.

**Methods:** First, we screened the entire Tox21 10K compound library using a qHTS KeratinoSens^TM^ (KS) assay and built a quantitative structure–activity relationship (QSAR) model based on the KS results. From the qHTS KS screening results, we prioritized 288 compounds to cover a wide range of structural chemotypes and tested them in the solid phase extraction–tandem mass spectrometry (SPE–MS/MS) direct peptide reactivity assay (DPRA), IL-8 homogeneous time-resolved fluorescence (HTRF) assay, CD86 and CD54 surface expression in THP1 cells, and predicted *in silico* sensitization potential using the OECD QSAR Toolbox (v4.5).

**Results:** Interpreting tiered qHTS datasets using a defined approach showed the effectiveness and efficiency of *in vitro* methods. We selected structural chemotypes to present this diverse chemical collection and to explore previously unidentified structural contributions to sensitization potential.

**Discussion:** Here, we provide a skin sensitization dataset of unprecedented size, along with associated tools, and analysis designed to support chemical assessments.

## Introduction

Allergic contact dermatitis (ACD) is a common environmental and occupational health problem. ACD is a delayed allergic reaction in the skin that comes into contact with a skin sensitizer (Kimber et al., 2011). The induction phase involves an endogenous protein reaction with haptens, dendritic cells (DCs) presenting the modified proteins as an antigen to naive T cells in the local lymph nodes, and the stimulation of specific T cells (Ainscough et al., 2013). Subsequent encounters with the same chemicals result in the elicitation phase, where memory T cells in the lymph nodes are activated and then recruit a specific T-cell-mediated immune response in the dermis (Meller et al., 2007). As the initial step in this immune process, the induction phase of skin sensitization is a key endpoint for product safety assessment.

The use of animal models for the evaluation of the sensitization potential of chemicals is being gradually phased out. In the recent decade, a mechanistic understanding of the causal relationship in skin sensitization allowed us to dissect the adverse effect to a series of biological events composed of molecule interactions. When it translates to risk assessment, a step-by-step evaluation of skin sensitization potential is based on these key events (KEs) which form the adverse outcome pathway (AOP), a structured representation. New alternative approaches are designed to detect these molecular and cellular signaling, which, when mapped to KEs in the AOP, have gained regulatory acceptance and are incorporated into AOP-based integrated testing strategies (Daniel et al., 2018; Hoffmann et al., 2018).

Currently, the skin sensitization OECD test guidelines are based on four KEs of the AOP: 1) covalent modification of self-proteins by sensitizers (haptenation), measured via, e.g., the direct peptide reactivity assay (DPRA) (OECD, 2019). The DPRA uses two synthetic peptides which contain a cysteine or a lysine residue to evaluate a chemical adduct formation ability with the thiol group and amine group, respectively (Gerberick et al., 2004; Gerberick et al., 2009); 2) antioxidant response element (ARE) assay monitors the activation of the Keap1/Nrf2 pathway, which plays a well-established role of indicating electrophiles, e.g., KeratinoSens^TM^ (KS) (Natsch et al., 2011; OECD, 2018a); 3) DC activation, which models the presentation of new antigens (hapten/carrier complexes); e.g., the human cell line activation test (hCLAT) (OECD, 2023) measures the induction of CD54 and CD86 in THP1 cells, which indicates the ability of a substance to activate and mobilize DCs in the skin (Sakaguchi et al., 2006; Nukada et al., 2012). Also pertaining to this KE, IL-8 production in THP1 cells following contact allergen stimulation was reported previously (Nukada et al., 2008). In contrast to the hCLAT, which detects the expression of cell surface markers, an IL-8 Luc THP1 cell line was used to quantify changes in IL-8 expression (OECD, 2016a), a cytokine associated with the activation of DCs (Takahashi et al., 2011); and 4) murine local lymph node assay (LLNA) measures lymphocyte proliferation, which represents the activation of the immune system (OECD, 2002). None of the *in chemico/in vitro* methods (KE 1–3) have been approved as stand-alone replacements for the LLNA (KE 4), which is historically the gold standard for assessing skin sensitization potential. To systematically evaluate and apply four *in chemico*/*in vitro* tests, i.e., the DPRA, KS, hCLAT, and IL-8 homogeneous time-resolved fluorescence (HTRF) (internal assay which maps to the same AOP KE as the hCLAT), we validated and adapted them into high-throughput screening (HTS) platforms and named them quantitative high-throughput screening (qHTS) KS, solid phase extraction–tandem mass spectrometry (SPE–MS/MS) DPRA, and CD86/CD54, and IL-8 HTRF assays accordingly. Among skin sensitization testing assays, only the qHTS KS assay was adapted to 1,536-well plates. Therefore, we first screened the Tox21 10K compound library in the KS assay and then performed follow-up testing in the other methods and generated hazard predictions using the OECD QSAR Toolbox (v4.5).

Machine learning classification models are computer algorithms capable of predicting categories for new unseen data based on annotated training data and have been widely applied to predict the skin sensitization potential (Wilm et al., 2018). A few validated QSAR models with defined applicability domains were developed to predict skin sensitization based on LLNA results or multiple non-animal tests (Lu et al., 2011; Alves et al., 2015; Hirota et al., 2015; Strickland et al., 2016; Braga et al., 2017; Strickland et al., 2017; Zang et al., 2017; Wilm et al., 2019). The feature sets for building these classification models included different combinations of *in vitro* assays (e.g., KS and hCLAT), *in chemico* assays (e.g., DPRA), and physicochemical properties (e.g., molecular weight and vapor pressure) (Wilm et al., 2018; Golden et al., 2020). Compared with other types of features, *in vitro* and *in chemico* assays can not only be used to predict the skin sensitization potential of a given compound with an unknown structure or physicochemical properties but also provide clues to understand the mechanisms of skin sensitization (Strickland et al., 2017; Zang et al., 2017). Since the Tox21 10K compound library is designed to cover a large chemical space (Bell et al., 2017), the composition of the compound library spans with a broad range of pharmaceuticals, pesticides, pharmaceutical aids, food additives, consumer products, industrial chemicals, cosmetics, household items, herbicides, and others. The KS dataset provides a valuable annotated dataset with diverse chemotypes that could be used to train machine learning classification models.

When integrating data from *in silico* and *in vitro* tests, regulators are faced with the question of whether non-animal test-generated information is reliable and predictive of the outcome in humans. Many studies have been conducted to validate the use of defined approaches (DAs) to satisfy specific regulatory needs (Rovida et al., 2015; Casati et al., 2018; Kleinstreuer et al., 2018). A DA consists of a fixed data interpretation procedure to interpret data generated with a defined set of information sources (e.g., *in silico, in chemico, in vitro*, and physico-chemical properties). For example, the 2-out-of-3 DA predicts a skin sensitization hazard by testing in up to three internationally accepted non-animal methods with OECD test guidelines (TGs) (DPRA, KS, and h-CLAT (Urbisch et al., 2015)). The sequential testing strategy (STS) (Nukada et al., 2013) is designed for sensitizing potency classification based on DPRA and hCLAT data and accepted in US EPA interim guidance but not included in OECD Guideline 497 due to the limited specificity against reference data (EPA, 2018). The integrated testing strategy (ITS) uses DPRA, hCLAT, and *in silico* predictions as data inputs (Takenouchi et al., 2015) and is included in OECD Guideline 497 (OECD, 2021). These DA predictions are rule-based, not influenced by expert judgment, which are published as case studies in OECD Guidance Documents (OECD, 2016b) for regulatory use (EPA, 2018; OECD, 2021). Therefore, the 2 out of 3, STS, and ITS DAs were applied to the set of compounds identified by the *in vitro* assays to characterize their predicted skin sensitization hazard and potency. Furthermore, we screened a large set of compounds in qHTS and *in silico* test batteries, trained a robust machine learning model for the KS assay, applied OECD-validated defined approaches to predict their skin sensitization potential, and applied cheminformatics techniques to characterize the results and yield structural insights into the skin sensitization potential.

## Materials and methods

### qHTS KS assay

The Nrf2/ARE-HaCaT-derived KS cell line was maintained in MEM supplemented with 10% FBS (HyClone), 100 U/mL penicillin, and 100 μg/mL streptomycin (Invitrogen). THP1 cells were cultured with RPMI 1640 Medium (Gibco) supplemented with 10% FBS (HyClone), 100 U/mL penicillin, and 100 μg/mL streptomycin (Invitrogen). The cells were maintained at 37°C under a humidified atmosphere in a 5% CO_2_ incubator.

KS cells were dispensed at 1,500 cells/5 μL/well in 1,536-well white wall/solid bottom plates using a Multidrop Combi (Thermo Fisher Scientific, Waltham, MA) dispenser. After the assay plates were incubated at 37°C/5% CO_2_ for 5 h, 23 nL of compounds dissolved in DMSO, a positive control (2,4-dinitrochlorobenzene), or DMSO only were transferred to the assay plate using a Wako Pintool station (Wako Automation, San Diego, CA). The chemicals including the positive control were tested in a 15-point concentration ranging from 2.8 nM to 92 µM. The plates were incubated at 37°C/5% CO_2_ for 23 h, followed by the addition of 1 μL/well of CellTiter-Fluor reagent into the assay plates using a BioRAPTR Flying Reagent Dispenser (Beckman Coulter, Brea, CA). After 1 h of incubation at 37°C/5% CO_2_, the fluorescence intensity was measured using a ViewLux (PerkinElmer, Shelton, CT) plate reader. Then, 4 μL/well of ONE-Glo was added using a BioRAPTR Flying Reagent Dispenser. After the plates were incubated at room temperature for 30 min, the luminescence intensity in the assay plates was measured using the ViewLux plate reader. A compound is considered positive when the induction of Nrf2/ARE luciferase is over 1.5-fold of the negative control when applying the OECD TG criteria. The compound with curve rank number >3 (defined in qHTS data analysis) is considered positive in curve rank criteria. The EC_1.5_ value represents the concentration for which the induction of luciferase activity is above the 1.5-fold threshold. I_max_ indicated the maximal activity value observed at any concentration of the tested chemical that was normalized to a percentage of 2-fold of the negative control value.

### IL-8 HTRF assay

THP1 cells were suspended in an assay medium (RPMI 1640 containing 10% FBS, 100 U/mL penicillin, and 100 μg/mL streptomycin) and dispensed at 2,000 cells/4 μL/well in a 1,536-well white-wall/solid-bottom plate using a Multidrop Combi (Thermo Fisher Scientific, Waltham, MA) dispenser. Then, 23 nL of compounds dissolved in DMSO, positive control lipopolysaccharides (LPSs, Sigma), or DMSO only were transferred to the assay plate using a Wako Pintool (Wako Automation, San Diego, CA). The final compound concentrations in the 4-µL assay volume ranged from 1.25 nM to 115 μM in 15 concentrations. The plates were incubated at 37°C/5% CO_2_ for 18 h, followed by the addition of 1 μL/well of pre-mixed anti-IL-8 antibodies (Cisbio, Bedford, MA) into the assay plates using a BioRAPTR Flying Reagent Dispenser. After incubation at room temperature for 2 h, the HTRF activity was the ratio of readings at channel 662 nm/615 nm, which was measured using an EnVision plate reader with the filter setting excitation 320 nm and emission 615/665 nm. Two criteria were used to identify a positive chemical in the assay were applied. The first criteria is based on curve ranking used in qHTS. Compounds with a curve rank number above 3 were considered active (curve fitting procedure defined in qHTS data analysis). The second criteria is based on, OECD TG 442E, which identifies a positive sensitizer based on the fold induction of luciferase activity over the vehicle control. A compound is considered positive when the induction of luciferase is at least 1.4-fold, and the lower limit of the 95% confidence interval is over 1.0-fold.

### SPE–MS/MS DPRA

A stock solution of the standard cysteine (Ac-RFAACAA-COOH) or lysine peptide (Ac-RFAAKAA-COOH) in traditional DPRA was mixed with an internal control alanine peptide (Ac-RFAAAAA-COOH) and diluted in PBS (pH = 7.5) or ammonium acetate (pH = 10.2), respectively, to obtain a final concentration of 5 µM of cysteine and 0.5 mM lysine peptide, as reported by Wei et al. (2021). By using lower peptide concentrations compared with OECD TG 442C, the high sensitivity of RapidFire-MS/MS and high reactivity of the thiol group in the cysteine peptide provide sufficient sensitivity for this qHTS DPRA method. Chemicals dissolved in DMSO with a concentration ranging from 0.25 μM to 1 mM, or 12 μM to 50 mM (DMSO final concentration is <0.1%), were added to the peptide mixtures and incubated at room temperature for 24 h in Nunc 384-well plates (Thermo Fisher). The compound–peptide mixtures were then analyzed by RapidFire-MS/MS. The RapidFire system, which conducts high-speed solid-phase extraction (SPE) (sampling, loading, washing, and injection) through multiple pumps and a valve system, delivers eluted analytes directly to the mass spectrometer (RapidFire-MS/MS) (Clausse et al., 2019). The RapidFire-MS/MS system was equipped with a C4 type A SPE cartridge. Agilent RapidFire 4.0 and Agilent MassHunter B.08.00 software were used for instrument control and data acquisition. Two criteria were used to identify a positive chemical in the assay. OECD Test Guideline 442C defines compounds depleting >13.89% cysteine peptide (DPRA-C) or depleting >6.38% the average of lysine peptide (DPRA-K) and cysteine peptide (DPRA-C) as active. In qHTS DPRA, the depletion % of the peptide is reflected as a negative range since the reduction in the peptide amount is the inhibition mode. Therefore, the chemicals with a curve rank less than −3 (inhibition mode: efficacy in the negative range) are positive in qHTS curve rank criteria.

### CD86 and CD54 surface expression measurement

Prior to the measurement of CD86 and CD54 surface expression, all compounds were prescreened for their non-cytotoxic concentrations using CellTiter Glo. Cell viability of each compound was tested in 1536-well white wall/solid bottom plates using a Multidrop Combi (ThermoFisher Scientific, Waltham, MA) dispenser. For compounds showing cytotoxicity at < 200 μM, the highest non-cytotoxic concentration was selected as the single concentration point to perform CD86 and CD54 measurement in flow cytometry. If a compound was not cytotoxic, the highest achievable concentration (100–200 µM) was selected as the test concentration. THP1 cells were seeded in a round-bottom 96-well plate at a density of 2 × 10^5^ cells/well and treated with chemicals for 24 h before harvesting. The cells were stained with 3 µL CD54 mAb (clone 6.5B4 from Agilent, DAKO, Santa Clara, CA) and 6 µL CD86 mAb (Biosciences, San Jose, CA) separately for 30 min at 4°C. All the samples were stained with live 760 (Biosciences). After washing and resuspending in PBS, flow cytometry analysis was performed in a iQue Plus flow cytometry system. The dead cells were gated out, and a total of 5,000 living cells were analyzed. The fluorescence intensity of each sample was normalized to as the relative fluorescence activity (percentage) of the positive control, 200 µM DNCB-treated group. OECD Test Guideline 442E defines compounds inducing CD86 > 1.5-fold or CD54 >2-fold above baseline (DMSO) as active in hCLAT, and the same threshold was applied here. Since compounds were tested in a single concentration in the CD86 and CD54 surface expression measurement, it was not possible to apply curve rank criteria for active call designation.

### qHTS data analysis

Data normalization and concentration–response curve fitting for the data from the qHTS screening and follow-up studies were performed in a 1,536-well plate, as previously described (Wang and Huang, 2016). In brief, qHTS KS and IL-8 HTRF assays, raw plate reads for each titration point, were normalized relative to the DMSO-only wells as follows: % activity = ((V_compound_–VDMSO)/VDMSO ×100, where V_compound_ denotes the compound well values and VDMSO denotes the median values of the DMSO-only wells (DMSO final concentrations ranging from 0.38% to 0.45%) and then corrected by applying an NCATS in-house pattern correction algorithm using compound-free control plates (i.e., DMSO-only plates) at the beginning and end of the compound plate stack (Wang et al., 2010). Concentration–response titration points for each compound were fitted to a four-parameter Hill equation yielding concentrations of half-maximal inhibitory activity (IC_50_) or half-maximal stimulatory activity (EC_50_) and maximal response (efficacy) values (Huang, 2016).

Compounds were designated as classes 1–4 according to the type of concentration–response curve observed (Inglese et al., 2006; Huang, 2016). Curve classes were further combined with efficacy and converted to curve ranks (Supplementary Table S1), which are numeric measures of compound activity ranging from −9 to 9 (Huang, 2016). Compounds that showed activation are assigned positive curve rank values, and compounds that showed inhibition are assigned negative values. Inactive compounds are assigned a curve rank of 0. Each compound was assigned an activity outcome, inactive, active, or inconclusive, based on the type of concentration–response curve and reproducibility (three independent runs), as described previously (Huang, 2016). For each assay run in multiple concentrations, chemicals with a curve rank below −3 or above 3 are considered actives. Activity calls were also applied based on criteria from the respective OECD guidelines, as described above. For assays with both OECD TG calls and curve rank calls (DPRA and KS), the two methods were assessed for concordance.

### Clustering and compound selection by chemotype

The active compounds from the Tox21 10K qHTS KS screen were analyzed for chemotype composition based on ToxPrint definitions. ToxPrint is composed of 729 uniquely defined chemical features coded in XML-based Chemical Subgraphs and Reactions Markup Language (CSRML), which were generated within the publicly available ChemoTyper application (https://chemotyper.org/) (Yang et al., 2015). Each compound was assigned to one chemotype cluster so that all compounds in that cluster share the same chemotype. As one compound could have multiple chemotypes, to select one chemotype for each compound, medium-sized chemotypes (shared by 10–20 compounds) were preferred over large chemotypes (shared by < 10 compounds) and over small chemotypes (shared by > 20 compounds). For small clusters with <3 compounds, compounds with AC_50_ < 10 μM and efficacy >50% were selected. Compounds were then selected from each chemotype cluster based on cluster size and primary KS assay activity. Chemotypes were sorted by cluster size-based order, and the top chemotype was then selected for each compound. The sorting and selection process starting with the most potent and efficacious compounds was selected from each cluster, and more compounds were selected from larger clusters. This process resulted in 288 compounds covering 141 representative chemotype clusters (column D in Supplementary Table S2), which were tested in offline qHTS KS assay again and then plated for follow-up testing in the SPE–MS/MS DPRA, CD86 and CD54, and IL-8 HTRF assays and prediction using the OECD QSAR Toolbox v4.5.

### Machine learning models

To model the KS assay results, the Tox21 10K KS dataset was downloaded from Tripod (https://tripod.nih.gov/tox/). The compound quality control (QC) results and additional annotations for each individual compound are publicly accessible at https://tripod.nih.gov/tox21/samples. The compounds that passed the QC test were split into two parts, approximately two-thirds of the dataset for model training and cross-validation, and the other one-third for external validation. Then, the desalted simplified molecular-input line-entry system (SMILES) structures were converted to ToxPrint fingerprints using ChemoTyper. The structure for each compound was represented as a bit vector where the presence or absence of the feature was recorded in a binary system as 1 or 0, respectively (Yang et al., 2015).

The ToxPrint fingerprints were used as input features to build a model to predict the KS assay results (active/inactive calls).

Five different classification algorithms were used, namely, Naïve Bayes (NB), neural networks (NNET), random forest (RF), support vector machine (SVM), and eXtreme Gradient Boosting (XGBoost). Models were built and tested using R version 3.4.2 with the “e1071” package for NB and SVM classifiers (Dimitriadou et al., 2005), “Random Forest” package for RF classifier (Liaw and Wiener, 2002), “nnet” package for NNET (Ripley, 2002), and “xgboost” package for the XGBoost classifier. The implementation of the NB classifier was adapted with the settings of Laplace smoothing, and the Gaussian Radial Basis Function kernel was used for the SVM classifier. In addition, the other parameters were set to default for the SVM, RF, and NNET classifiers. The optimal constant parameters for the XGBoost classifier were as follows: control the learning rate (0.01), maximum depth of a tree (3), and subsample ratio of columns when constructing each tree (0.5).

Models were built using the best practices (Tropsha, 2010). In brief, feature selection (on the training set only) as a preprocessing step prior to modeling was performed using four methods, namely, Fisher’s exact test with *p*-value, area under the receiver operating characteristic curve (AUC-ROC) value, importance scores from XGBoost (gain score), and RF (Gini score) algorithm. Fisher’s exact test evaluates the enrichment of every feature in the active compounds. Features were selected at five different *p*-value cutoffs ranging from 0.01 to 0.05 with an interval 0.01. The AUC-ROC method evaluates every feature for its predictive value of the endpoint being modeled. An AUC-ROC value was calculated for every feature. Features were selected at five different AUC-ROC cutoffs ranging from 0.52 to 0.60 with an interval of 0.02 using “pROC” packages (Sing et al., 2005; Robin et al., 2011). The “xgboost” (Chen et al., 2015) and “Random Forest” (Liaw and Wiener, 2002) packages were applied to retrieve feature importance scores. Features ranked with gain or Gini scores were picked at 10 intervals from the top 10 to top 50. After feature selection, models for the qHTS KS assay endpoint were built using the selected feature subsets via supervised machine learning.

To evaluate model performance, the dataset was randomly divided into 70% (4,564 compounds) as a training set and 30% (1956 compounds) as a test set. Each model was evaluated by the internal 3-fold cross-validation on the training set. To ensure the robustness of our results, each training model was repeated 20 times. Due to the imbalanced class distributions of active and inactive compounds, the training set was rebalanced using four different subsampling methods, namely, downsampling, upsampling, random oversampling examples (ROSE), and the Synthetic Minority Oversampling TEchnique (SMOTE) via the “ROSE” and “DMwR” packages in R (Torgo and Torgo, 2013; Lunardon et al., 2014). Model performance on the test set was measured by the AUC-ROC, balanced accuracy (BA) using the “ROCR” and “pROC” package (Sing et al., 2005; Robin et al., 2011), and Matthews correlation coefficient (MCC) using the “mltools” package in R. The plots were generated using the “ggplot2” package in R. Representative structures containing any of these significant features were drawn using the ChemDraw Professional software (version 17.1).

### OECD QSAR Toolbox automated workflow prediction

The 288 compounds identified by the application of chemotype clusters to the primary KS screen were run through the OECD QSAR Toolbox v4.5 automated workflow to predict the skin sensitization potential (Yordanova et al., 2019). In brief, the SMILES specifications of the chemical structure for each compound were used as inputs. Skin sensitization hazard predictions (positive or negative) were made using the automated workflow for “EC3 from LLNA or skin sensitization from Guinea Pig Maximization Test (GPMT) for defined approaches (SS AW for DASS).” If the automated workflow could not make a prediction because the compound was a salt, the salt was dissociated, and the automated workflow was applied to the organic portion of the compound to make a hazard prediction. The QSAR Toolbox does not make skin sensitization hazard predictions for inorganic substances.

### Application of defined approaches

While none of the OECD-validated *in vitro* or *in chemico* assays are considered adequate to predict the skin sensitization potential as stand-alone methods, data generated with tests addressing multiple KEs of the skin sensitization AOP can be used together, as well as with information sources such as *in silico* and read-across predictions from chemical analogs, within DAs. The first OECD guideline on DAs for skin sensitization was recently approved (OECD, 2021) and includes multiple rule-based DAs that provide predictions of potential dermal sensitization hazard classification and potency category. These validated DAs were extensively characterized and shown to either provide the same level of information or be more informative than the murine LLNA (OECD TG 429) for hazard classification (i.e., sensitizer versus non-sensitizer) and for Globally Harmonized System of Classification and Labeling of Chemicals (GHS) potency subcategorization (Secretariat, 2015). The “2o3” DA provides a prediction for hazard classification based on majority concordant results of the DPRA, KS, and hCLAT, and the integrated testing strategy “ITSv2” DA predicts the GHS potency category (1A, 1B, or not classified [NC]) based on the DPRA, hCLAT, and OECD QSAR Toolbox *in silico* prediction. An additional DA for hazard prediction, the STS, was included in an interim science policy published by the US EPA (EPA, 2018) as an alternative to the animal test. These three DAs were applied here to chemicals with the requisite information sources based on the OECD TG criteria.

## Results

### qHTS KS screen and model performance

The qHTS KS primary screening tested ∼8,300 unique compounds at 15 concentrations ranging from 0.04 nM to 92 μM in three independent runs. To evaluate qHTS performance, a set of parameters were calculated, as described previously (Huang, 2016). For primary screening, the signal-to-background (S/B) ratio was 2.89 ± 0.22, the coefficient of variation (CV) was 4.66% ± 1.16%, and the average Z′ factor was 0.76 ± 0.03. The cell viability assay was measured simultaneously in the same well with KS. For the cell viability assay, the S/B ratio was 166.80 ± 4.64, CV was 7.18% ± 0.68%, and Z’ factor was 0.79 ± 0.03. The qHTS reproducibility parameters given in Supplementary Table S3 showed that the three independent runs reproduced well, and nearly no mismatch was found. As described in the section on data analysis, each compound activity from three independent runs was defined as an active, inconclusive, or inactive based on curve classes. Most of the compounds (75.74%) were inactive in qHTS KS assay. The 676 active compounds and 5,844 inactive compounds which served to balance the dataset were selected for qHTS KS computational modeling.

**FIGURE 1:**
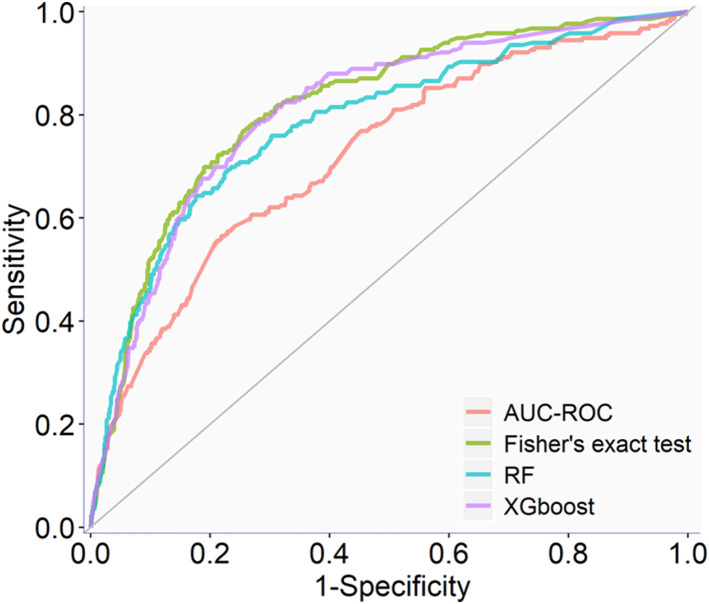
Example ROC curves of the optimal QSAR models on the external validation dataset.

The full qHTS KS assay dataset (a total of 6,520 compounds given in Supplementary Table S4) generated from screening the Tox21 10K compound library was split into two parts, approximately two-thirds for model training and cross-validation and one-third for external validation, as described in Materials and methods. AUC-ROC, BA, and MCC were used to measure model performance, which exhibited the same trend (Table 1). The cross-validation results showed that the optimal models for each feature selection method all achieved good performances with AUC-ROC value >0.69 (Table 1). The overall best model achieved an AUC-ROC of 0.81 ± 0.01, which was generated by a combination of Fisher’s exact test with *p*-value ≤0.05 (feature selection method), RF (machine learning algorithm), and upsampling (rebalancing strategy). The optimal models from each feature selection method were employed to predict the external validation dataset. The external validation AUC-ROC values ranged from 0.73 to 0.81 (Figure 1), and the optimal parameter combination that produced the best external validation AUC-ROC is consistent with the cross-validation results given in Table 1 (i.e., Fisher’s exact test with *p*-value ≤0.05, RF algorithm, and upsampling). Fisher’s exact test evaluates the enrichment of every feature in the active compounds and showed the best performance in four statistical tests. The chemotype features with *p*-value <0.05 using Fisher’s exact as a feature selection are given in Supplementary Table S5.

**TABLE 1:** Optimal classification performance (AUC-ROC, BA, and MCC values) of each feature selection method for predicting KS using a cross-validation of training set results.

Feature selection method	Machine learning algorithm	Rebalancing method	Cutoff	AUC-ROC	BA	MCC
AUC-ROC	NB	ROSE	0.52	0.72 ± 0.03	0.69 ± 0.02	0.27 ± 0.02
AUC-ROC	NNET	Original	0.52	0.70 ± 0.03	0.68 ± 0.02	0.28 ± 0.03
AUC-ROC	RF	Upsampling	0.52	0.76 ± 0.02	0.72 ± 0.02	0.32 ± 0.03
AUC-ROC	SVM	Upsampling	0.52	0.74 ± 0.02	0.70 ± 0.02	0.29 ± 0.03
AUC-ROC	XGBoost	Original	0.52	0.76 ± 0.02	0.71 ± 0.02	0.31 ± 0.03
Fisher’s exact test	NB	Original	0.01	0.78 ± 0.01	0.73 ± 0.01	0.33 ± 0.02
Fisher’s exact test	NNET	ROSE	0.05	0.74 ± 0.02	0.70 ± 0.02	0.28 ± 0.04
Fisher’s exact test	RF	Upsampling	0.05	0.81 ± 0.01	0.75 ± 0.01	0.35 ± 0.02
Fisher’s exact test	SVM	Upsampling	0.03	0.78 ± 0.01	0.73 ± 0.01	0.32 ± 0.02
Fisher’s exact test	XGBoost	Upsampling	0.05	0.79 ± 0.01	0.73 ± 0.01	0.32 ± 0.03
RF	NB	Original	40	0.74 ± 0.02	0.69 ± 0.02	0.28 ± 0.03
RF	NNET	Upsampling	50	0.69 ± 0.02	0.66 ± 0.01	0.23 ± 0.03
RF	RF	Original	50	0.77 ± 0.02	0.71 ± 0.02	0.32 ± 0.04
RF	SVM	Upsampling	50	0.73 ± 0.02	0.69 ± 0.02	0.28 ± 0.04
RF	XGBoost	Original	50	0.75 ± 0.02	0.70 ± 0.02	0.30 ± 0.04
XGBoost	NB	Original	50	0.76 ± 0.02	0.71 ± 0.02	0.30 ± 0.03
XGBoost	NNET	Original	40	0.72 ± 0.02	0.69 ± 0.02	0.29 ± 0.03
XGBoost	RF	Original	50	0.79 ± 0.02	0.74 ± 0.02	0.35 ± 0.04
XGBoost	SVM	Upsampling	50	0.76 ± 0.02	0.71 ± 0.02	0.30 ± 0.02
XGBoost	XGBoost	Upsampling	50	0.77 ± 0.02	0.71 ± 0.02	0.30 ± 0.02

### Comparison among *in vitro* and *in silico* results

**FIGURE 2:**
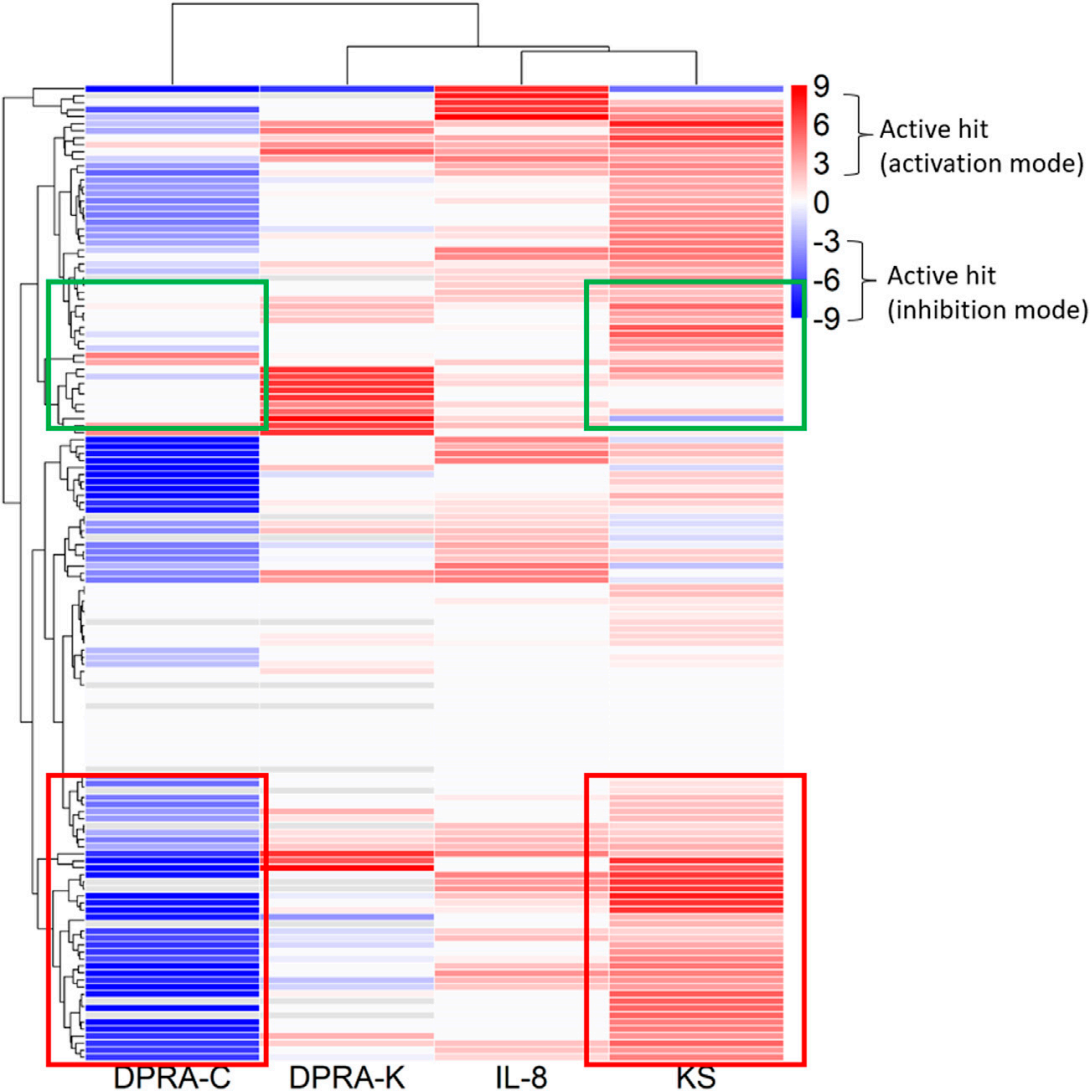
Schematic charts for screening, chemical prioritization workflow and qHTS data interpretation.

As shown in Figure 2, after the primary screen of the Tox21 10K compound library in qHTS KS assay, a total of 288 compounds were selected based on primary qHTS KS assay activity and chemotype diversity and then tested in SPE-MS/MS DPRA, CD86 and CD54 surface expression, and IL-8 HTRF assays. The original *in vitro* skin sensitization test battery assays were optimized to qHTS testing to accommodate the compound plate, which are in 1,536- or 384- or 96-well plates. The difference in the testing conditions and threshold for positive/negative calls of each assay is given in Table 2 accordingly. The key limitation is the fixed test compound concentration (up to 100–200 µM) which impacts the sensitivity of CD86 or CD54 surface expression.

**TABLE 2:** Comparison of assay conditions of skin sensitization *in vitro* assays and their qHTS versions.

	Culture time	Exposure time	Maximal test concentration	Negative/positive threshold
KS	24 h	48 h	5,000 mg/L	1.5-fold induction
qHTS KS	5 h	24 h	92 µM	Curve rank number >3 in curve rank criteria
				1.5-fold induction in OECD TG criteria

	C/K-peptide concentration	Exposure time	Test method	Prediction model
DPRA-Cys	0.667 mM in pH 7.5 phosphate buffer	24 h	HPLC	Negative: Cys % depletion <13.89%
				Low reactivity: 13.89 < Cys % depletion <23.09%
				Moderate reactivity: 23.09 < Cys % depletion <98.24%
				High reactivity: Cys % depletion >98.24%
SPE–MS/MS DPRA-C	0.5 µM in pH 7.5 phosphate buffer	24 h	MS-MS	Same as above
DPRA-Lys	0.5 mM in pH 10.2 ammonium acetate buffer	24 h	HPLC	Negative: mean Cys and Lys % depletion <6.38%
				Low reactivity: 6.38% < mean Cys and Lys % depletion <22.62%
				Moderate reactivity: 22.62% < mean Cys and Lys % depletion <42.47%
				High reactivity: 42.47% < mean % depletion <100%
SPE-MS/MS-DPRA-K	0.5 mM in pH 10.2 ammonium acetate buffer	24 h	MS-MS	Same as above

	Culture time	Exposure time	Test concentration	Negative/positive threshold
hCLAT-CD86	24 h	24 h	Up to 5,000 mg/L	RFI 150
CD86	24 h	24 h	Up to 100 µM	RFI 150
hCLAT-CD54	24 h	24 h	Up to 5,000 mg/L	RFI 200
CD54	24 h	24 h	Up to 100 µM	RFI 200

	Cell line	Exposure time and test concentration	Assay type	Negative/positive threshold
IL8-Luc	THP1-Luc	24 h, 20 mg/mL	Luciferase reporter assay	1.5-fold
HTRF IL8	THP1	16 h, up to 100 µM	HTRF immune assay	1.5-fold

Positive/negative outcomes of each compound in different assays based on the curve rank criteria qHTS and OECD TG criteria are given in Supplementary Table S6. The concordance of the activity calls based on the two criteria for qHTS KS assay was 86% in balanced accuracy, and for SPE-MS/MS DPRA assay, it was 90% in balanced accuracy; the comparison of activity calls is given in Supplementary Table S7. OECD TG criteria have more positive calls than the curve rank criteria. This is because the curve rank has an additional criterion, curve class, to measure the dose–response curve quality based on the curve fitting. The curve rank criteria are more suitable for qHTS data since the curve class values the data confidence as an important factor in large-scale data interpretation.

The same set of chemicals was also run through the OECD QSAR Toolbox DASS automated workflow, which provides the prediction of skin sensitization hazards based on a read-across approach from a large reference database of animal and human data. The concordance of active and inactive outcomes between each of the non-animal test methods and with OECD QSAR Toolbox predictions is shown in active/inactive concordance tables (Tables 3, 4). The active or inactive outcome shown in Table 3 was based on the curve rank number (0 is inactive; >3 or < −3 as active), and Table 4 was based on the OECD TGs criteria (described in methods of each assay). The number of concordant active and inactive hits is shown in the column and row intersections; the higher numbers indicate better concordance of the assay pairs. SPE–MS/MS DPRA-C (cysteine depletion) and SPE–MS/MS DPRA-K (lysine depletion) were evaluated separately in terms of curve rank criteria. When considering the concordance of compounds with active outcomes in SPE–MS/MS DPRA-C/K, we used OECD TG 442C criteria to make the call. Overall, qHTS KS and SPE–MS/MS DPRA have the highest active correlation among all *in vitro* assays regardless of different active/inactive outcome criteria. When comparing *in vitro* assays with OECD Toolbox predictions, DPRA-C demonstrated the highest concordance of actives based on the curve rank criteria, and DPRA-C/K was most concordant with the OECD Toolbox when using OECD TG criteria for actives.

**TABLE 3:** Concordance of qHTS KS, SPE–MS/MS DPRA-C, SPE–MS/MS DPRA-K, and IL-8 HTRF assays (based on the curve rank criteria) and OECD Toolbox automated workflow predictions for 288 chemicals. CD86/CD54 surface expression is not included for curve rank criteria because it was tested at a single concentration. The compounds detected as active and inactive assays were counted separately. The gray area is for concordance of compounds detected as active in assays. The blue area is for concordance of compounds detected as inactive in assays.

Curve rank criteria	Number of chemicals detected as active in assays				
Method	qHTS KS (117)	SPE–MS/MS DPRA-C (128)	SPE–MS/MS DPRA-K (8)	IL-8 HTRF (52)	OECD TB*(174)
qHTS KS (171)		60	1	24	80
SPE–MS/MS DPRA-C (149)	97		8	28	88
SPE–MS/MS DPRA-K (269)	158	149		1	6
IL-8 HTRF (236)	143	128	221		33
OECD TB* (32)	23	19	30	27	
	Number of chemicals detected as inactive in assays				

*71 out of domain chemicals are not included.

**TABLE 4:** Concordance of qHTS KS, SPE–MS/MS DPRA-C/K, IL-8 HTRF, and CD86/CD54 surface expression assays (based on OECD TG criteria) and OECD Toolbox automated workflow predictions for 288 chemicals. The compounds detected as active and inactive were counted separately. The gray area is for concordance of compounds detected as active in assays. The blue area is for concordance of compounds detected as inactive in assays.

OECD TG criteria	Number of chemicals detected as active in assays				
Method	qHTS KS (143)	SPE–MS/MS DPRA-C/K (146)	CD86/CD54	IL-8 HTRF (81)	OECD TB*(174)
qHTS KS (145)		84	11	45	96
SPE–MS/MS DPRA-C/K (131)	80		14	52	100
CD86/CD54 (239)	130	123		6	11
IL-8 HTRF (207)	109	105	177		51
OECD TB*(32)	21	18	27	26	
	Number of chemicals detected as inactive in assays				

*71 out of domain chemicals are not included.

**FIGURE 3:**
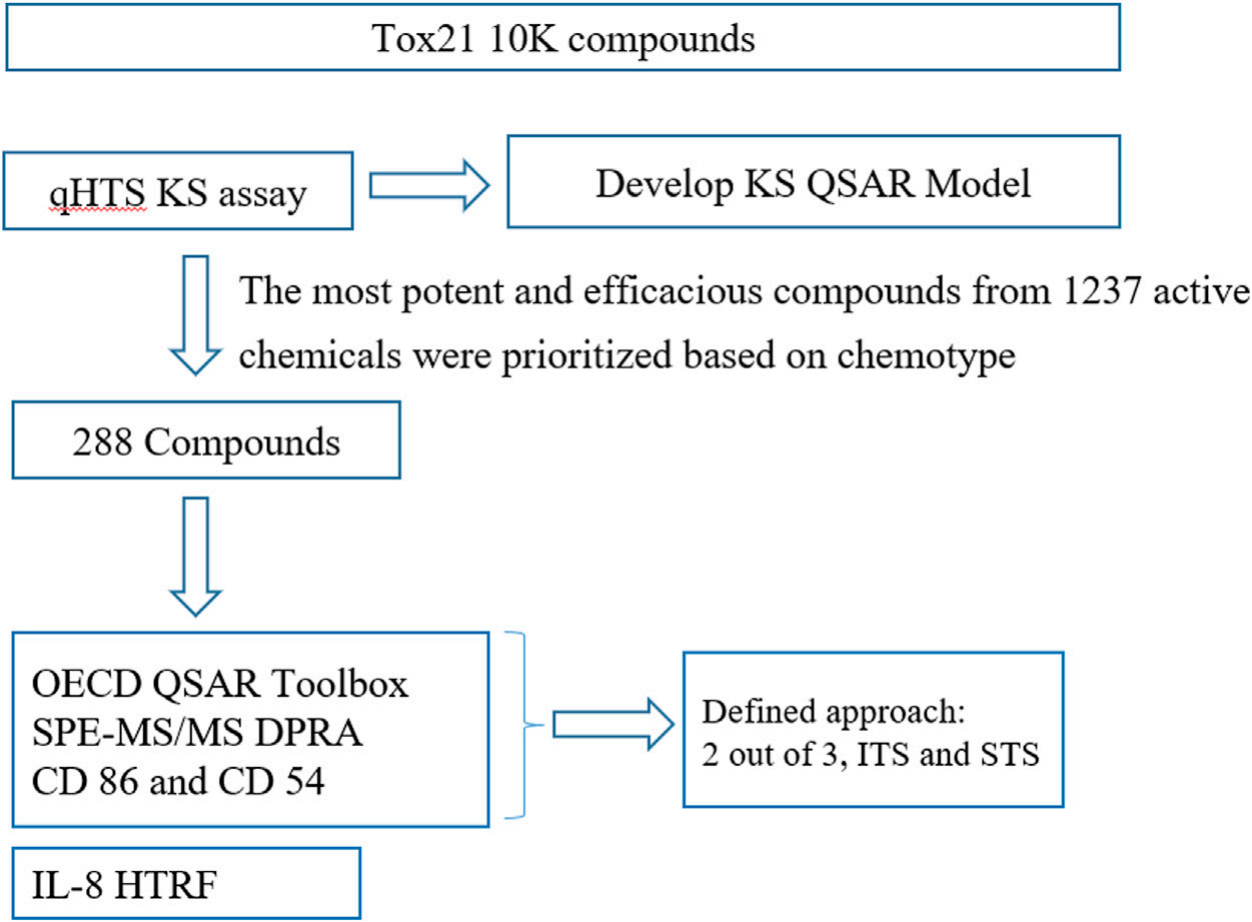
Activities of chemotypes found in compounds tested in qHTS KS, SPE–MS/MS DPRA-C, SPE–MS/MS DPRA-K, and IL-8 HTRF assays. The average curve rank number of qHTS KS, SPE–MS/MS DPRA-C, SPE–MS/MS DPRA-K, and IL-8 HTRF assays was plotted against the chemotypes assigned to the 288 compounds.

The 288 chemicals were described by chemotypes to study structural alerts, as described in Clustering and compound selection by chemotype, and the representative chemotype is shown in Supplementary Table S2 column D. The distribution is shown as a chemical count in each chemotype in Supplementary Table S8. The curve rank is a measurement of compound activity based on the curve class and efficacy, as described in Materials and methods and Supplementary Table S1. To visualize different chemotype activities in each assay, the average curve rank of chemicals containing a representative chemotype in KS, DPRA-C, DPRA-K, and IL-8 assays was plotted against the activities in qHTS KS, SPE–MS/MS DPRA-C, SPE–MS/MS DPRA-K, and IL-8 HTRF assays (Figure 3). The raw data are shown in Supplementary Table S9. The gradient red and blue color indicates the range of the average curve rank number (9 to −9). SPE–MS/MS DPRA-C and SPE–MS/MS DPRA-K are inhibition mode assays, so dark blue (−9) means that most of the peptides are depleted. Bright red (9) indicates agonists in qHTS KS and IL-8 HTRF assays. Due to the single-concentration test condition of CD86 and CD54 surface expression (limited by test concentration), the results were not shown in the heat map. The threshold in OECD TG 442E (OECD, 2023) to define a positive is that a compound increases by 2-fold the fluorescence intensity of DMSO for CD54 or by 1.5-fold for CD86. We marked those compounds with an asterisk (*) after the compound name in Supplementary Table S6. In Figure 3, the qHTS KS assay detects 54 chemotypes (117 chemicals) as actives, and the SPE–MS/MS DPRA-C assay detects 65 chemotypes (126 chemicals) as actives. The common active chemotypes in both qHTS KS and SPE–MS/MS DPRA-C are highlighted in column A of Supplementary Table S9. Although the qHTS KS assay found fewer actives than SPE–MS/MS DPRA-C, most of the chemotypes, which were only active in qHTS KS assay, were negative in the OECD Toolbox, except chemotype “ring:hetero_[3]_O_epoxide” and “bond:S(=O)O_sulfuricAcid_generic which are positive in qHTS KS only.” When clustered by chemotypes in the dendrogram, the branch of the dendrogram labeled with the red box indicates a better correlation between SPE–MS/MS DPRA-C and qHTS KS assays. The chemotypes in the branch of the dendrogram labeled with the green box were mostly actives in qHTS KS but not in SPE–MS/MS DPRA-C.

### Defined approaches for predicting the sensitization potential

**FIGURE 4:**
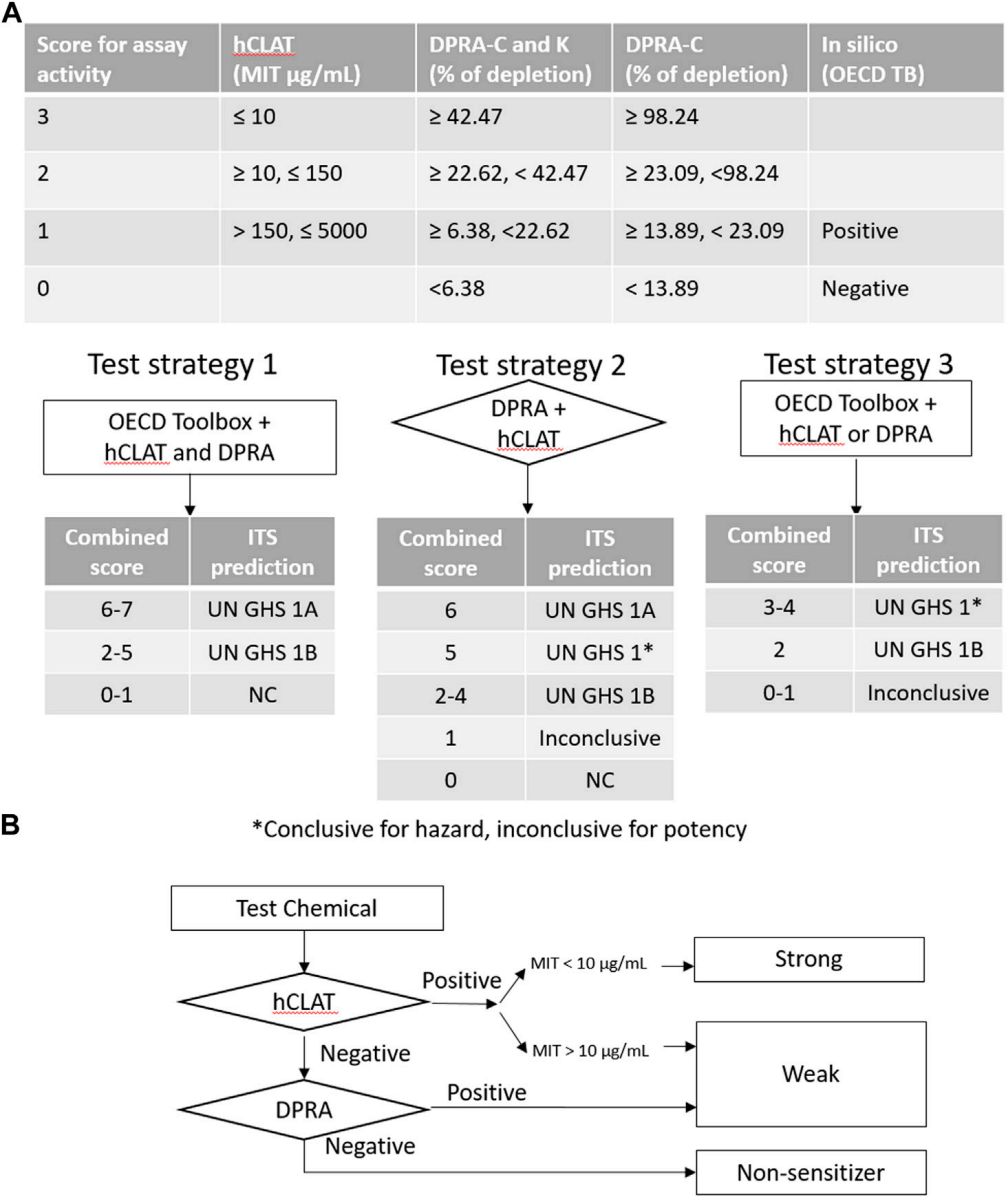
Decision tree for categorizing sensitization potency in defined approaches. **(A)** ITS is a score-based system used to evaluate different test strategies. It requires multiple tests conducted before the result interpretation. The sum of the score of assay activity or/and predicted outcome determines the GHS category. **(B)** STS is a fixed stepwise approach to decide the sensitization potency. The flowchart provides the designation of sensitization potency based on hCLAT and DPRA.

**FIGURE 5:**
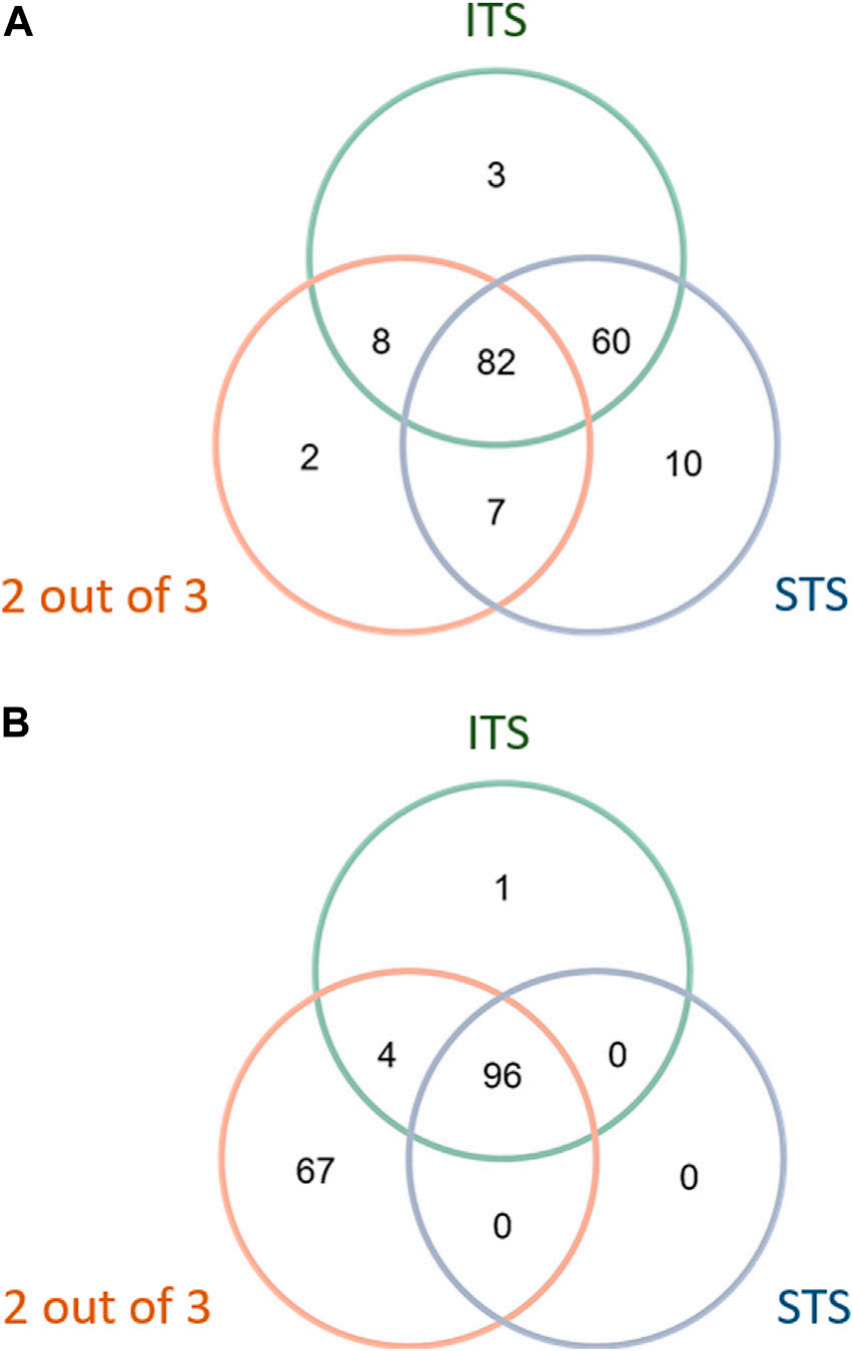
Concordance of hazard predictions in 2 out of 3, STS, and ITS DAs. **(A)** Positive concordance among DA hazard predictions. **(B)** Negative concordance among DA hazard predictions.

**FIGURE 6:**
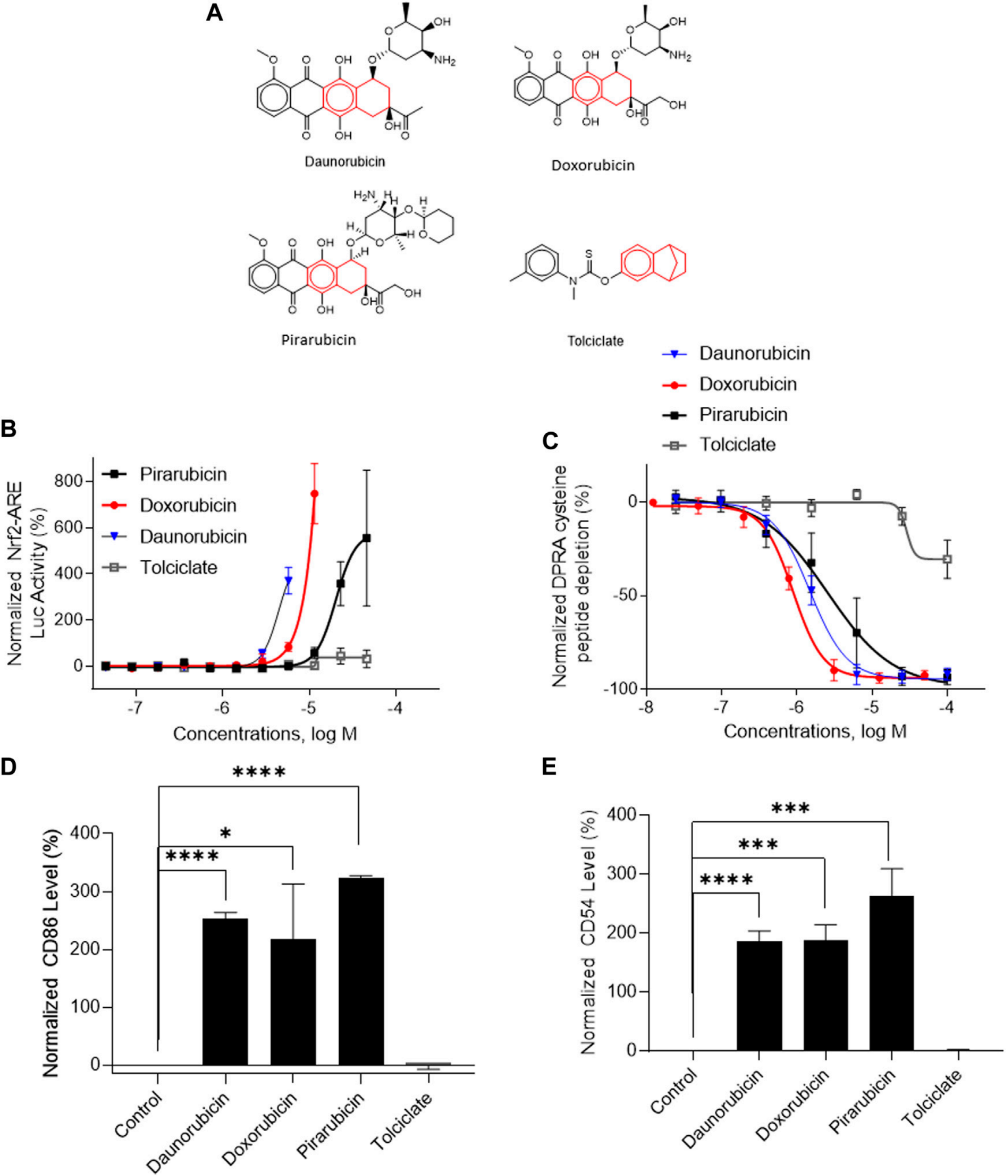
*In vitro* assay results of compounds containing chemotype “ring:fused_[6_6]_tetralin.” **(A)** Structures of daunorubicin, doxorubicin, pirarubicin, and tolciclate. **(B)** DPRA-C assay data were normalized to the percentage activity of positive control MDG. **(C)** KS assay data were normalized to the activity of positive control DNCB. **(D)** CD86 expression level was normalized to the percentage activity of 1.5-fold of the DMSO vehicle control. The asterisk * indicates significantly different from DMSO control in Student’s t-test (*n* = 3). **(E)** CD54 expression level was normalized to the percentage activity of 2-fold of the DMSO vehicle control. The data were expressed as the mean ± SD from three independent experiments. The asterisk * indicates significantly different from the DMSO control in Student’s t-test (*n* = 3).

Three DAs (2o3, ITSv2, and STS) with varying degrees of regulatory acceptance were applied to the chemical set tested here. The *in chemico, in vitro,* and *in silico* information sources were combined as outlined in the data interpretation procedures in OECD GL 497 (i.e., 2o3, ITS) or the EPA Interim Science Policy (i.e., STS). The data interpretation decision trees for the ITS and STS are shown in Figures 4A, B accordingly. The results are shown in Venn diagrams as positive concordance (Figure 5A) and negative concordance (Figure 5B), and DA calls of each compound are shown in detail in Supplementary Table S6 (column AK–AO). Across the three DAs that provide hazard predictions, 82 chemicals were positive in all the DAs, 157 chemicals were positive in at least two DAs, and 172 chemicals were positive in at least one DA (Figure 5A). A total of 96 chemicals were consistently predicted to be negative across the DAs, with an additional 72 predicted negative by 2o3 and/or ITS (majority by 2o3 only) (Figure 5B). Potency predictions provided by the ITS and STS DAs were extremely consistent across the tested set, with 82% agreement between the two approaches (Table 5). Consistent predictions from both ITS and STS DAs yielded 96 chemicals predicted as NC, 133 chemicals predicted as GHS 1B, and 6 chemicals predicted as GHS 1A. One chemical predicted as GHS 1B by ITS was predicted as GHS 1A by STS. Twelve chemicals deemed inconclusive and five NC in the ITS DA were predicted as GHS 1B by the STS. Among the six compounds which were predicted as GHS 1A compounds by both DAs, some were substantiated by the literature (daunorubicin) or are identified here for the first time as potential strong sensitizers (pirarubicin, 1,4-dihydroxy-2-naphthoic acid). Particularly, three GHS 1A compounds (doxorubicin, daunorubicin, and pirarubicin) were assigned with the chemotype “ring:fused_[6_6]_tetralin” which has the highest ITS DA score among all the chemotypes (structures shown in Figure 6A). There are four chemicals with this chemotype. Daunorubicin, doxorubicin, and pirarubicin are GHS 1A, and only tolciclate is GHS 1B. Although these chemicals tested positive in KS and DPRA, the potency difference of GHS 1A and GHS 1B is clearly separated, as shown in Figures 6B, C. Tolciclate is inactive in qHTS KS (Figure 6B) and CD86/CD54 surface expression assays (Figures 6D, E) and showed weak depletion in the SPE–MS/MS DPRA-C assay (Figure 6C).

**TABLE 5:** Concordance of STS and ITS DA-predicted sensitization potency based on the GHS category.

	DA STS			
DA ITSv2		GHS NC	GHS 1B	GHS 1A
	GHS NC	96	5	0
	GHS 1B	0	133	1
	GHS 1	0	12	0
	GHS 1A	0	2	6

### Selected chemicals to represent chemotype features with the skin sensitization potential

**FIGURE 7:**
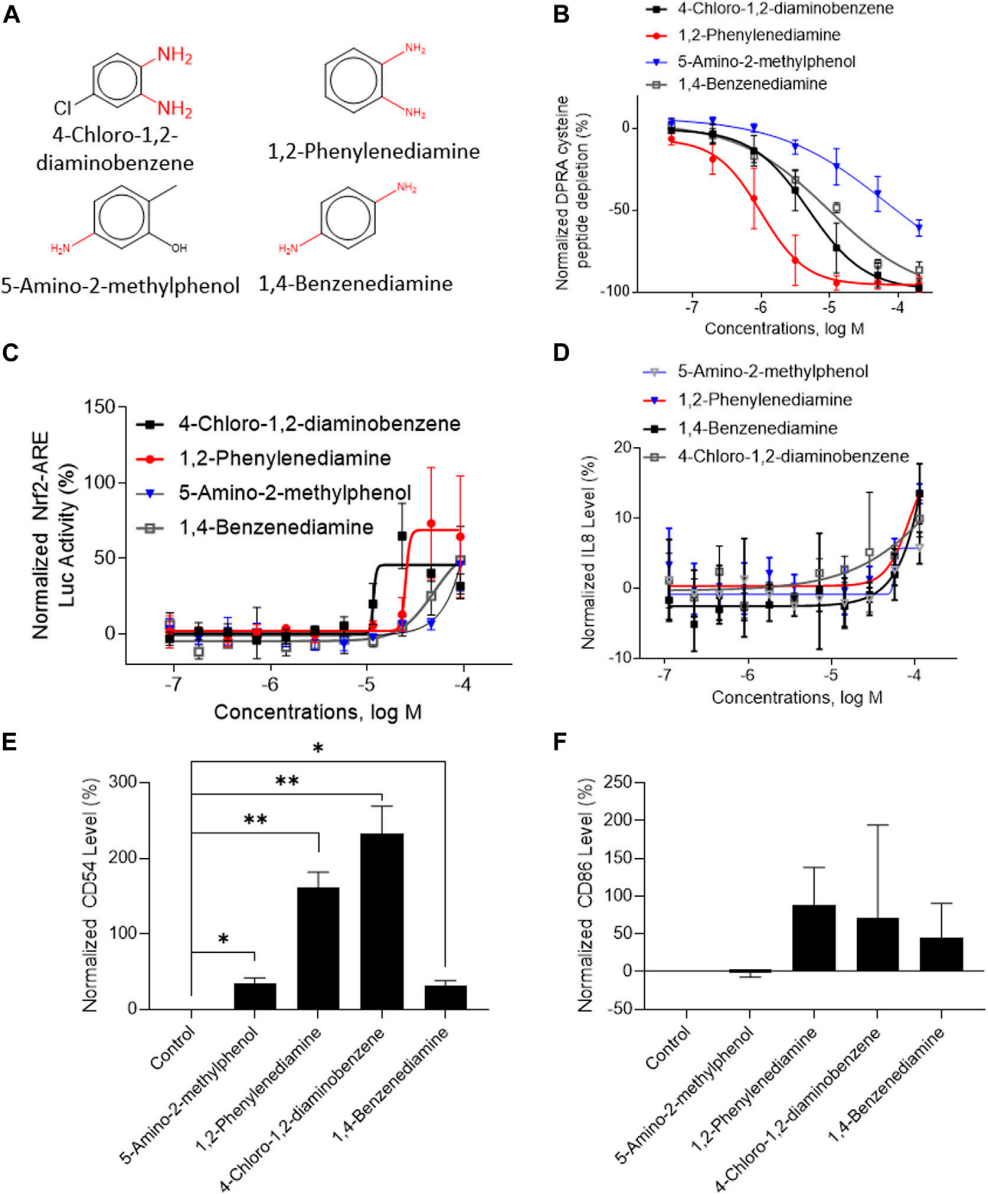
*In vitro* results of compounds containing chemotype “bond:CN_amine_pri-NH2_aromatic.” **(A)** Structure of 4-chloro-1,2-diaminobenzene, 1,2-phenylenediamine, 5-amino-2-methylphenol, and 1,4-benzenediamine. **(B)** SPE–MS/MS DPRA-C assay data were normalized to the percentage activity of positive control MDG. **(C)** qHTS KS assay data were normalized to the activity of positive control DNCB. **(D)** IL-8 protein level was normalized to the percentage activity of 2-fold of the DMSO vehicle control. **(E)** CD54 expression levels were normalized to the percentage of 1.5-fold of DMSO vehicle control. The asterisk * indicates significantly different from the DMSO control in Student’s t-test (*n* = 2). **(F)** CD86 expression levels were normalized to the percentage of 2-fold of the DMSO vehicle control. The asterisk * indicates significantly different from the DMSO control in Student’s t-test (*n* = 2). The data were expressed as the mean ± SD from three independent experiments.

**FIGURE 8:**
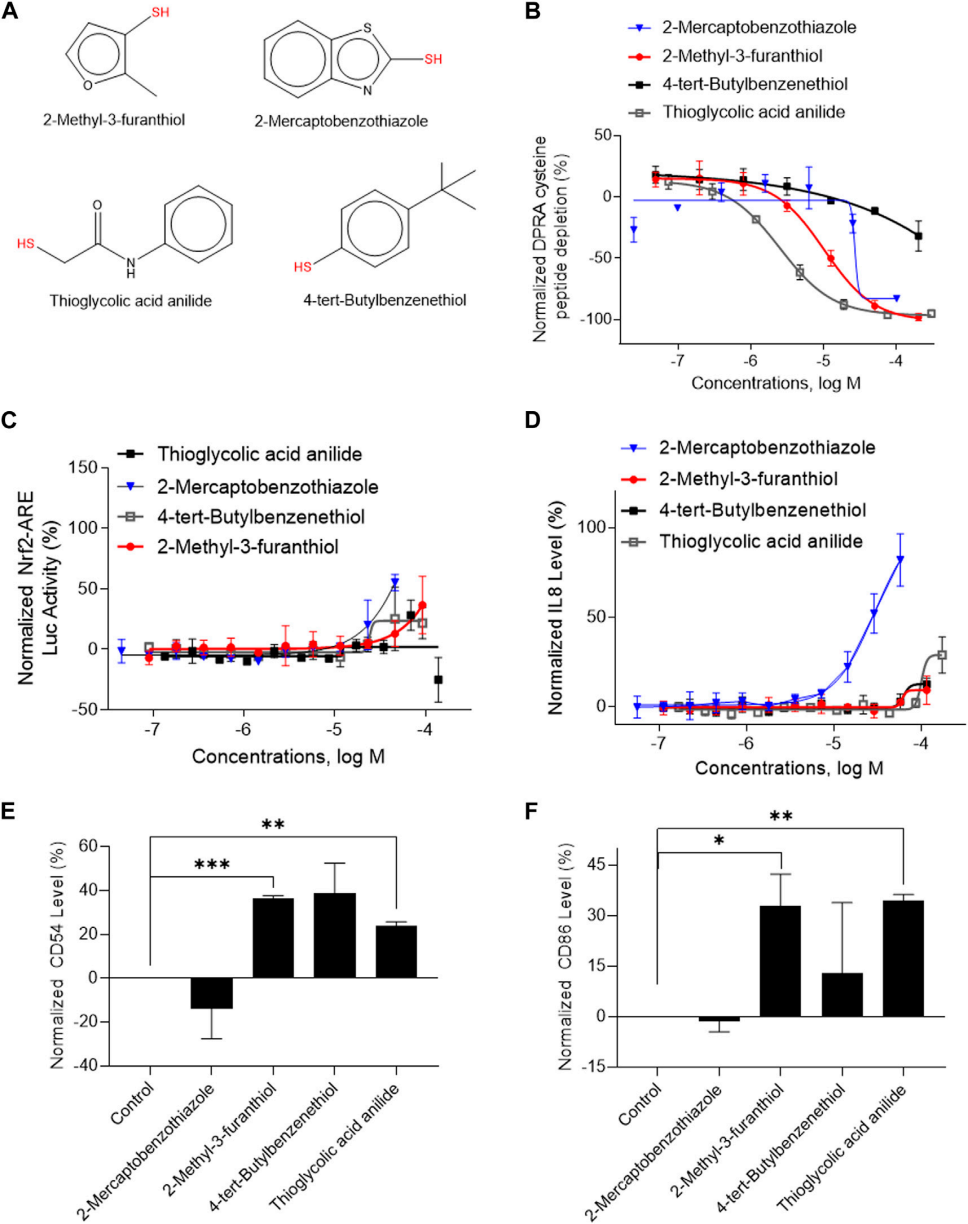
*In vitro* results of compounds containing chemotype “bond:QQ (Q∼O_S)_sulfhydride.” **(A)** Structure of 2-methyl-3-furanthiol, 2-mercaptobenzothiazole, thioglycolic acid anillide, and 4-chloro-1,2-diaminobenzene. **(B)** SPE–MS/MS DPRA-C assay data were normalized to the percentage activity of positive control MDG. **(C)** qHTS KS assay data were normalized to the activity of positive control DNCB. **(D)** IL-8 protein level was normalized to the percentage activity of 2-fold of the DMSO vehicle control. **(E)** CD 54 expression level was normalized to the percentage activity of 1.5-fold of the DMSO vehicle control. The asterisk * indicates significantly different from the DMSO control in Student’s t-test (*n* = 2). **(F)** CD86 expression levels were normalized to the percentage activity of 2-fold of DMSO vehicle control. The asterisk * indicates significantly different from DMSO control in Student’s t-test (*n* = 2). The data were expressed as the mean ± SD from three independent experiments.

A total of 141 representative chemotypes (column D in Supplementary Table S2) are assigned to the 288 chemicals. We presented two chemotypes associated with most of the chemicals that were positive in two assays of three key events (SPE–MS/MS DPRA, qHTS KS, and IL-8 HTRF or CD86/CD54 surface expression). The chemotypes “bond:CN_amine_pri-NH2_aromatic” and “bond:QQ(Q∼O_S)_sulfhydride” represent electrophiles which are well-known structural alerts of sensitizers. The structures of these chemicals are shown in Figures 7A, 8A (representative chemotype highlighted in red). There are four chemicals in each chemotype. All four chemicals in the “bond:CN_amine_pri-NH2_aromatic” were active in SPE-MS/MS DPRA-C and qHTS KS assays (Figures 7B, C). Although all were inactive in the IL-8 HTRF assay, a small increase in the IL-8 expression level was observed at the highest concentration (Figure 7D). 1,2-Phenylenediamine and 4-chloro-1,2-diaminobenzene were active in the CD54 and CD86 surface expression assay (Figures 7E, F). The compounds represented by the chemotype “bond:QQ (Q∼O_S)_sulfhydride” are 2-methyl-3-furanthiol, 2-mercaptobenzothiazole, 4-tert-butylbenzenethiol, and thioglycolic acid anilide, which showed depletion in the SPE-MS/MS DPRA-C assay (Figure 8B) but a relatively marginal induction of the ARE gene in the qHTS KS assay (Figure 8C). They also induced IL-8 protein expression (Figure 8D) or CD54/CD86 surface expression in THP1 cells (Figures 8E, F).

## Discussion

A number of non-animal alternative methods have been recently evaluated for regulatory use to assess substances for skin sensitization potential. OECD has adopted nine non-animal methods addressing mechanisms under the first three KEs of the skin sensitization AOP. No single assay is sufficient to fulfill regulatory requirements on the skin sensitization potential and potency of chemicals comparable to that provided by the regulatory animal tests. This study used a tiered strategy to evaluate the Tox21 10K compound library by using OECD-recommended *in vitro* tests to first screen the full chemical library (qHTS KS) and perform follow-up testing (SPE–MS/MS DPRA, IL-8 HTRF, and CD86/CD54 surface expression) for the skin sensitization potential. The prioritized chemicals were predicted by the DASS automated workflow in the OECD QSAR Toolbox and three DA strategies (2 out of 3, ITS, and STS), and the full qHTS KS dataset was used to build a machine learning model. This study provides a novel skin sensitization dataset on a wide range of chemicals, delivers an *in silico* tool that can be used for structure-based screening, and serves to demonstrate the benefits of integrating different elements in chemical safety assessment.

Since the qHTS platform performed assays in miniaturized wells, we adapted the OECD TG *in vitro* assays to achieve the best assay performance. There are a number of technical considerations that go along with adapting the assays to the qHTS format. The qHTS KS assay was performed in a 1,536-well plate with a co-measurement of cell viability. The simultaneous measurement of the reporter gene and cell viability ensures monitoring of cytotoxicity of each compound at all tested concentrations. SPE–MS/MS DPRA was measured using the RapidFire-MS/MS method in a 384-well plate. Compared to HPLC-based DPRA, the RapidFire-MS/MS DPRA renders high sensitivity and efficiency, which also lowers peptide usage concentrations to 5 µM. The RapidFire-MS/MS method was validated with known sensitizers and highlighted by the high accuracy because of an internal control alanine peptide (Wei et al., 2021). The CD86 and CD54 surface expression assay was conducted in a single non-toxic concentration due to the highest test concentration range limit in qHTS (the highest test concentration of nine common chemicals with OECD DASS is below hCLAT MIT). That’s because compounds from the Tox21 10K compound library were prepared as 10–20 mM stock in DMSO, which limits the highest test concentration to 100–200 µM in hCLAT assays. The cytotoxic compounds are tested at the highest non-cytotoxic concentration for CD54/CD86 activation. If a compound shows no cytotoxic effect or CD54/CD86 activation at the highest achievable concentration, the compound is classified as negative for sensitization. Inevitably, some false negatives are most likely due to the concentration limit. The hCLAT assay needs to be run in a higher concentration range in the future. In this study, the IL-8 protein expression was measured by an HTRF-based technology instead of a luciferase reporter assay. It largely reduces the background signal compared with the IL-8 luciferase reporter assay (Takahashi et al., 2011). The IL-8 luciferase reporter assay and hCLAT were previously reported to be similar because they assess the same KE in the AOP (Wilm et al., 2018). Even with test concentration limits, we found that the IL-8 HTRF assay detects more positives than CD86 and CD54 surface expression and is more sensitive most of the time. The modifications in qHTS KS, SPE–MS/MS DPRA, and IL-8 HTRF assay protocols would not impact data interpretation since all assays are monitored with negative and positive controls, and the results are applied to OECD TG criteria, as well as qHTS curve rank criteria to define the assay call. Curve rank criteria weight more on reproducible curves and tend to prioritize compounds with high efficacy and potency. Generally, the curve rank criteria display a higher bar to define a chemical as positive because the original design is for potent drug candidate selection.

In addition to the qHTS-adapted *in chemico* and *in vitro* techniques, we also leveraged *in silico* methods in this study. The Tox21 10K compound library is designed to cover a large regulatory relevant chemical space, and the qHTS KS assay screen on the Tox21 chemicals represents the largest skin sensitization dataset ever generated. These data were used to train and test a robust QSAR model that demonstrated good performance metrics when applied to the test set, such as an AUC-ROC of 0.81, a BA of 0.75, an MCC of 0.30, a sensitivity of 0.63, and a specificity of 0.86 for the optimal model. Based on the cross-validation results, the optimal models for each feature selection method all achieved good performances with AUC-ROC values >0.69, BA > 0.66, and MCC >0.23 (Table 1). The overall best model achieved an AUC-ROC of 0.81 ± 0.01 with a BA of 0.75 ± 0.01 and an MCC of 0.35 ± 0.02, which was generated by a combination of Fisher’s exact test with *p*-value ≤0.05 (feature selection method), RF (machine learning algorithm), and upsampling (rebalancing strategy). Consistent with our previous report on constructing and optimizing classification models for predicting skin toxicity endpoints (Xu et al., 2020), the optimal model performance also depended on a combination of the specific machine learning algorithm, rebalancing strategy, and feature selection method (Table 1). The key structural features are widely applicable for predicting the bioactivity of a given compound (Xu et al., 2023a; Xu et al., 2023b). In this study, the chemical features with *p* value < 0.05 were associated with skin sensitization (Supplementary Table S5), and some of them have literature support. For example, the feature “ring:hetero_[5_6]_N_S_benzothiazole_(1_3-)” was found significant for skin sensitization (*p*-value = 6.34 × 10^−8^) in this study. Consistent with this finding, benzothiazole and benzotriazole derivatives, widely used as industrial chemicals (e.g., plasticizers), are closely associated with many biological effects such as skin sensitization (Carlsson et al., 2022). The feature “bond:COH_alcohol_aromatic_phenol” was found significant for skin sensitization (*p*-value = 7.76 × 10^−12^) in this study. Consistent with this finding, chemicals with this chemotype such as hydroquinone, 5-chlorosalicylanilide, and 5-amino-2-methylphenol have been reported to cause skin sensitization in LLNA (Kern et al., 2010; Basketter et al., 2014). In the future, without performing KS in a wet laboratory, this QSAR model based on the Tox21 10K data in this study can be used as a prescreening step. The model code is publicly available via GitHub (https://github.com/TX-2017/machine-learning/blob/main/R_code.R) and will be part of future updates to the Integrated Chemical Environment (https://ice.ntp.niehs.nih.gov). The complete code package and the training dataset are accessible for any researcher who is interested in predicting the skin sensitization of other compounds. It is important to note that our predictive model was constructed based on the results obtained from HTS of the KS assay, so it can only be used to predict the skin sensitization potential of compounds as indicated by the KS assay alone. While the KS assay serves as a guideline for skin sensitization in regulatory decision making, the HTS version we utilized does not adhere to OECD guidelines. Additionally, it is crucial to highlight that the predictive results necessitate experimental validation.

The existing *in silico* tool we applied was the OECD Toolbox DASS automated workflow, which uses a read-across approach based on animal data to predict the skin sensitization potential. We compared SPE–MS/MS DPRA, qHTS KS, CD86/CD54 surface expression, and IL-8 HTRF assay results with OECD Toolbox DASS automated workflow prediction. Among these assays, DPRA and KS detected most actives compared to OECD Toolbox prediction. This was expected because SPE–MS/MS DPRA and qHTS KS assays depend largely on chemical reactivity, which aligns with QSAR-based automated flow prediction. qHTS KS and SPE–MS/MS DPRA-C assays both evaluate a chemical’s reactivity with the thiol group. Most of the well-characterized sensitizers have electrophilic reactivity, so it was also expected that qHTS KS and SPE–MS/MS DPRA-C assays have similar active distribution patterns of chemotype activity (Figure 3). It was interesting that the KS assay detects more actives than the DPRA-C assay in the curve rank criteria. For example, the chemicals with chemotypes in the green box (Figure 3) activated KS but showed no activity in the DPRA-C assay. For example, four chemicals, 5-azacitidine, ouabain, proscillaridin, and digoxigenin in the chemotype “bond:COH_alcohol_diol_(1_3-),” induce Nrf2-ARE gene expression without cytotoxicity at low concentrations (positive in both OECD TG and curve rank criteria when only considering the KS assay outcome). However, they start to show cytotoxicity at higher concentrations (curve rank number −7 to −9 in the cytotoxicity assay) (Supplementary Table S6).

The DAs combine results from biological endpoint *in vitro* and/or *in silico* predictions in a rule-based judgment to predict the sensitization potential of compounds. Here, we used assays that align to the AOP and OECD QSAR Toolbox automated workflow predictions to apply the 2 out of 3, STS, and ITS DA strategies. The decision trees of ITS and STS strategies are shown in Figure 4. The 2 out of 3 needs DPRA, KS, and hCLAT assay results, which require more test systems than STS and ITS, and has the potential drawback of misclassifying weak sensitizers as non-sensitizers. DA 2 out of 3 provided 67 negative calls, which disagreed with ITS and STS (Figure 5B). A common reason for this mismatch was that a compound showing strong peptide depletion (>23.09%) in the DPRA assay but negative in qHTS KS and CD86/CD54 surface expression assays would be classified as a non-sensitizer by the 2 out of 3 but not by the ITS or the STS. This is amplified by the low sensitivity of qHTS assays of CD86/CD54 due to the limited concentrations tested in qHTS assays. In this study, DA ITS and STS showed strong concordance in GHS potency categorization (Table 5), which was unsurprising since they are based on two of the same *in vitro* assays. STS tends to classify marginal effects to sensitization, while ITS has a buffering category for inconclusive decisions based on the decision tree in GL 497. We found that 5 STS-predicted GHS 1B sensitizers were ITS-predicted non-sensitizers because they are negative in CD86/CD54 surface expression and weakly depleted peptides in DPRA. There were nine chemicals in common with the OECD DASS database (Supplementary Table S10). When comparing the qHTS version with traditional guideline assay result in DASS, the qHTS DPRA peptide assay and qHTS KS detect six out of eight and seven out of nine positives in traditional DPRA and KS assay, respectively. Only three out of nine chemicals showed marginal CD86 or CD54 activation because their hCLAT MIT is higher than the highest test concentration in this study. When it comes to the defined approach, the concordance of our ITS and STS for hazard classification with that of the OECD DASS was 8/9 and 7/9, accordingly. The concordance with OECD DASS results was 7/9 for the 2o3 DA. However, ITS and STS potency categorization from the OECD DASS was not concordant with our qHTS ITS and STS approaches, which was 1/9 for ITSv2 and 5/9 for STS. The low-potency concordance is due to the missing hit in the hCLAT assay. Additionally, this paper does not consider the borderline ranges since OECD TG 497 determined the borderline ranges based on the log pooled median absolute deviation from ring trial laboratory test results. However, the deviation of the qHTS assay result is already counted in the evaluation of concentration–response curve reproducibility (curve classification and curve class rank number).

We used several compounds to show the benefits of sequentially performing *in vitro* assays based on ITS or STS DA strategies. For example, the compounds shown in Figures 7, 8 are all predicted as sensitizers in the OECD QSAR Toolbox automated workflow. In the ITS DA strategy, performing the DPRA assay alone is often sufficient to determine the sensitization hazard potential. These compounds all showed depletion activity in the SPE–MS/MS DPRA-C assay (Figures 7B, 8B). In this way, these compounds could be identified as sensitizers in the most cost-efficient way. If aiming to categorize by potency, first performing the CD86/CD54 surface expression assay can determine the potency of these sensitizers when applying the STS DA strategy. This is especially true for a situation with known structural alerts of chemicals which are expected to react with DPRA peptides. For example, the chemicals with chemotype feature “bond:CN_amine_pri-NH2_aromatic” (Figure 7) have primary amine and chemotype “bond:QQ (Q∼O_S)_sulfhydride” (Figure 8) have a thiol group. The compounds in these two chemotypes showed activity in IL-8 HTRF or CD54 or CD86 assays. Due to the concentration limits in the qHTS platform, the marginal effect at the current tested concentration in hCLAT could not be performed at a higher concentration. However, we confirmed their activity in IL-8 assay. If run in a traditional hCLAT, they could be identified as GHS 1B. Indeed, among them, 2-mercaptobenzothiazole, 1,2-phenylenediamine, and thioglycolic acid anilide were reported as strong sensitizers in humans (McCord, 1946; Kligman, 1966; Nalluri and Williams, 2014). Therefore, IL-8 HTRF is a more sensitive assay and can be used under the condition which has a limitation of achieving a higher concentration of the test article.

LLNA is the gold standard of the skin sensitization animal test, and LLNA only correctly predicts 70%–80% human skin allergic response in the human repeat insult patch test (HRIPT) (ICCVAM, 2011). Among the 288 chemicals tested in the current study, 15 were reported with LLNA results, 16 were reported with HRIPT results, and 4 chemicals were tested in both HRIPT and LLNA methods (Supplementary Table S11). DA 2 out of 3 is a hazard identification process instead of potency prediction compared to STS and ITSv2. The sensitivity and specificity analysis of DA 2 out of 3 with LLNA and HRIPT shows that DA 2 out of 3 is more accurate in predicting HRIPT (sensitivity: 62.5%; specificity: 75%) rather than LLNA (sensitivity: 46.2%; specificity: 100%).

Here, we screened a large set of compounds using *in vitro* qHTS and *in silico* test batteries, applied OECD-validated defined approaches (2o3 and ITSv2) and STS published in the EPA Interim Science Policy to predict their skin sensitization potential, and used a subset of the data to train a machine learning model for KS assay. The current study provides a valuable dataset (Supplementary Table S4) with diverse chemicals that associated chemotypes with assay activities. The successful implementation of OECD *in vitro* test battery assays into the qHTS platform renders the opportunity to rapidly generate more useful datasets for *in silico* modeling in the future. The machine learning-based QSAR model provides an *in silico* tool to predict the KS assay activity. Even though some assays have a relatively low hit rate partly due to the limited test concentrations in this platform, the use of various assays in the skin sensitization test battery combined with automated workflow prediction in the DA strategy provided predictions of skin sensitizer hazard and potency.

## Data Availability

The original contributions presented in the study are included in the article/Supplementary Material; further inquiries can be directed to the corresponding authors.
